# Case Report: Successful treatment of metastatic retinoblastoma with CNS involvement with anti-GD2 immunotherapy, intrathecal topotecan and reduced systemic chemotherapy

**DOI:** 10.3389/fped.2024.1509645

**Published:** 2025-01-17

**Authors:** Cristina Larrosa, Margarida Simao-Rafael, Noelia Salvador, Juan Pablo Muñoz, Cinzia Lavarino, Guillermo Chantada, Jaume Mora

**Affiliations:** ^1^ Oncology Department, SJD Pediatric Cancer Center Barcelona, Hospital Sant Joan de Deu, Barcelona, Spain; ^2^ Laboratory of Molecular Oncology, Pediatric Cancer Center Barcelona, Hospital Sant Joan de Déu, Barcelona, Spain; ^3^ Scientific Director, Laboratori de Tumors del Desenvolupament, Institut de Recerca Sant Joan de Deu, Barcelona, Spain

**Keywords:** metastatic retinoblastoma, anti-GD2 immunotherapy, naxitamab, GD2, intrathecal topotecan

## Abstract

High-dose chemotherapy with autologous stem cell rescue has improved outcomes in patients with metastatic retinoblastoma (RB). Despite significant advances, acute and long-term side-effects, particularly in visually impaired and cancer-predisposed patients, underscore the need for additional treatment options. Monoclonal antibodies (mAbs) directed against the tumor-associated antigen GD2 are of considerable interest. Additional lines of RB research include tracking minimal disseminated disease (MDD) to permit timely intervention in patients with CNS metastasis. We present two cases of bilateral, metastatic RB, managed with the anti-GD2 mAb naxitamab following reduced intensity myeloablative chemotherapy and autologous stem cell transplant (ASCT) with intrathecal topotecan for MDD detected in the CSF. The patients remain disease-free 10 and 9 years after initial diagnosis. While additional studies are needed, the results suggest anti-GD2 mAbs and CNS-directed chemotherapy may improve long-term outcomes and reduce cytotoxicity for high-risk patients with RB.

## 1 Introduction

Retinoblastoma (RB) is a highly invasive tumor and the most common primary intraocular malignancy in children. It arises from the developing retina, which originates from the neuroectoderm, a tissue responsible for forming the central nervous system (CNS) (1). The disialoganglioside GD2 is overexpressed in tumors of neuroectodermal origin, including RB and neuroblastoma (NB) (2). GD2 plays an important role in malignant transformation and is a well-established therapeutic target (3).

**Table 1:** F, female; D, day.

Category	Case 1	Case 2
Diagnosis	Bilateral intraocular RB	Bilateral intraocular RB
Gender, age at diagnosis	Female/5 months	Female/12 months
Stage	Left eye: C; right eye: C	Left eye: E; right eye: C
Germline RB1 mutation	p.Ala74Glufs*4	p.Gln354*/c.1060C > T
Local Eye Treatment	– Left eye: Intravitreal topotecan (30 μg), transpupilar cryotherapy. Enucleation due to suboptimal response. – Right eye: intra-arterial melphalan (4 mg) and topotecan (1 mg).	– Left eye: enucleation upfront. – Right eye: Intravitreal topotecan (30 μg), intra-arterial melphalan (4 mg) and topotecan (1 mg). Enucleation due to retinal detachment and neovascular glaucoma.
– Systemic treatment (1st line)	– Carboplatin (500 mg/m2) (switched to cisplatin 100 mg/m2 due to allergy) etoposide (100 mg/m2), vincristine (1.5 mg/m2) – Cyclophosphamide (65 mg/kg/day), idarubicin (10 mg/m2/day) and vincristine (1.5 mg/m2).	– Carboplatin (500 mg/m2), etoposide (100 mg/m2), vincristine (1.5 mg/m2) (15 cycles)
Relapse	7 years of age; CNS*, bone and liver.	5 years of age; right eye, CNS* and BM.
MDD at relapse	– BM: negative cytology, negative CRX mRNA. – CSF: negative cytology, positive CRX mRNA.	BM: positive cytology, positive CRX mRNA. CSF: negative cytology, positive CRX mRNA.
Salvage Treatment	– Intrathecal topotecan (0.4 mg, 6 cycles) – Ifosfamide (3 g/m2/day, 3 days), doxorubicin (37.5/mg/m2/day, 2 days) – High dose chemotherapy – WITHOUT carboplatin: – Thiothepa (300 mg/m2/day, 1 day), etoposide (250 mg/m2/day, 3 days) and autologous stem cell rescue – Naxitamab (3 mg/kg/day, days 1, 3, 5) with sargramostim (500 µg/m²/day, days 0-5).	– Intrathecal topotecan (0.4 mg, 6 cycles) – Induction regimen as per COG ARET 0321: Vincristine (1.5 mg/m2, D1, D8) and D15, cyclophosphamide (1950mg/m2, D1), and etoposide (120 mg/m2/day, D1-2). Carboplatin omitted. – High dose chemotherapy without carboplatin: Thiothepa (300 mg/m2/day, 1 day), etoposide (250 mg/m2, 3 days) and autologous stem cell rescue. – Naxitamab (3 mg/kg/day, days 1, 3, 5) with sargramostim (500 µg/m²/day, days 0–5).
Outcome	– Complete remission. Follow up: 4 years after metastatic relapse.	– Complete remission. – Follow up: 5 years after extraocular relapse.
Functional outcome	– Grade 4 bilateral visual loss. – Grade 2 tubulopathy.	– Grade 3 hearing loss (secondary to chemotherapy). – Total visual loss.

*CNS relapse considered by minimal detectable disease (MDD).

Anti-GD2 monoclonal antibodies (mAbs) have significantly improved outcomes in high-risk NB (4–10). Naxitamab, a humanized version of m3F8 (hu3F8), received FDA breakthrough designation in 2018 and full approval in 2020. It is indicated, in combination with GM-CSF, for pediatric and adult patients with high-risk NB in the bone or bone marrow who demonstrate partial response, minor response, or stable disease following standard induction therapy (11). This approval was based on data from the pivotal phase II trial (Study 201, NCT03363373), which evaluated naxitamab in patients with high-risk NB refractory to initial standard treatments or demonstrated insufficient response to therapies for progressive or relapsed disease (12).

In high-income countries offering timely diagnosis and specialized care, RB overall survival rates have reached 95%, however, survival rates are significantly lower in low-income countries (13, 14). Treatment of metastatic RB presents formidable challenges, with intensive, multimodal regimens associated with significant toxicities (15). Metastatic disease frequently involves regional lymph nodes, bone and bone marrow (BM) and the CNS (16). CNS metastasis resulting from optic nerve infiltration is associated with a dismal prognosis (17).

In a recent study of patients with metastatic RB treated with high-dose chemotherapy and autologous hematopoietic stem cell transplant (HDC-ASCT), overall survival for patients with metastatic disease outside of the CNS was 76.7%, decreasing to less than 10% for those with CNS involvement (18). The treatment of RB with CNS metastasis lacks effective systemic agents that can penetrate the blood brain barrier (BBB). However, intrathecal topotecan has improved long-term survival in patients with metastatic CNS disease (19, 20).

Complications of metastatic RB and associated treatments include toxic deaths, hearing loss, neurocognitive impairment, and second malignant neoplasms (SMNs) (21–24). Germline cancer-predisposing mutations, present in 40% of RB patients, heighten the risk of SMNs (1). In high-income settings, metastatic retinoblastoma often arises after unsuccessful attempts at eye-preserving treatments in patients who have undergone extensive prior therapies. This further underscores the need for alternative, less toxic treatment options.

Here, we report two cases of metastatic RB managed with the anti-GD2 mAb naxitamab as consolidation after reduced-intensity chemotherapy and ASCT. In both cases the detection of CRX (cone-rod-homeobox) in the cerebrospinal fluid (CSF) prompted the use of CNS targeted therapy with intrathecal topotecan. Despite the historically challenging prognosis of systemic and CNS relapsed RB (25–27), both patients are alive and disease-free 3 years after the metastatic relapse.

## 2 Cases description

A summary of the cases information can be found in Table 1.

### 2.1 Case 1

**Figure 1:**
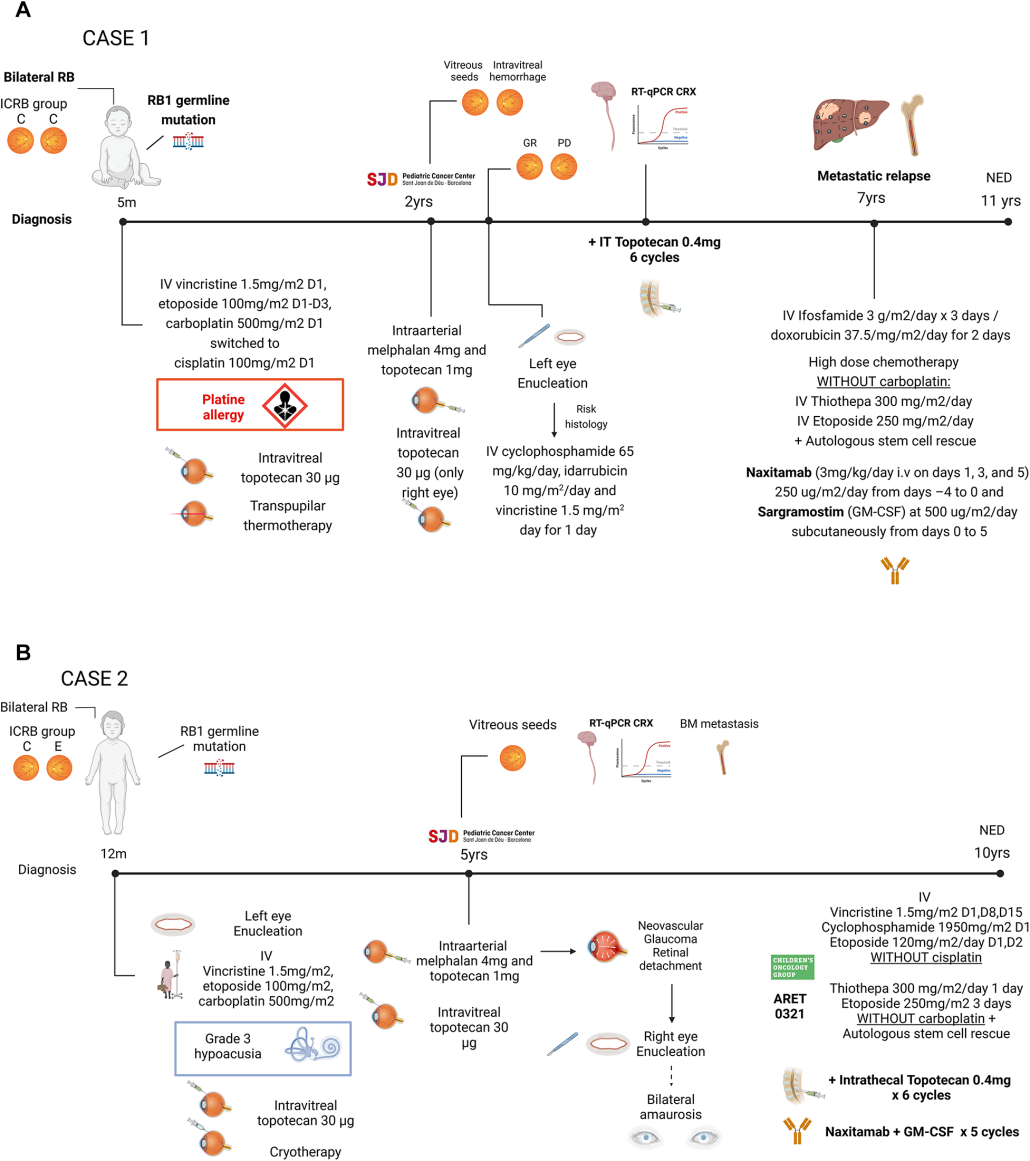
**(A)** ICRB, international classification of retinoblastoma; IV, intravenous; IT, intrathecal. **(B)** IV, intravenous; IT, intrathecal; NED, no evidence of disease. Created in BioRender.

The first patient was diagnosed with bilateral group C RB at 5 months of age, as per international classification of intraocular RB International Classification for Intraocular Retinoblastoma (ICRB). The initial treatment included an undetermined number of cycles of systemic vincristine, cisplatin, and etoposide (28, 29) causing grade 3 allergy to both cisplatin and carboplatin (See Figure 1A for additional details). Information regarding the patient's response to this initial treatment is not available. At 2 years of age, the patient arrived at our institution with active bilateral intraocular RB, subsequently confirmed to carry an RB1 germline mutation. Six tandem intra-arterial chemotherapy cycles were administered, yielding a favorable response in the right eye but suboptimal results in the left eye, requiring enucleation. Histological analysis of the enucleated eye revealed disease spread to the ciliary body and sclera, prompting adjuvant therapy with systemic alkylating agents, anthracyclines, and vincristine. Disease evaluation showed CRX mRNA positivity in the CSF along with normal cytology and no CRX detection in the BM (30–32). Intrathecal topotecan (0.4 mg) was added to systemic chemotherapy for 6 total monthly doses. After the first cycle, CRX mRNA status became negative and remained negative throughout subsequent cycles.

Four years later, the patient presented with metastatic lesions in the BM, bone and liver. Rescue treatment comprised two cycles of ifosfamide and doxorubicin, followed by reduced-dose of myeloablative therapy (etoposide and thiotepa, no carboplatin due to prior allergy) and ASCT, achieving complete remission (CR). Because of reduced myeloablative therapy, 5 cycles of naxitamab (3 mg/kg/day i.v on days 1, 3, and 5) 250 ug/m2/day from days –4 to 0 and sargramostim (GM-CSF) at 500 ug/m2/day subcutaneously from days 0 to 5 (33) were added for further consolidation (34).The patient experienced no major acute toxicities, but reported grade 2 pain, hypotensiona and urticarial reaction.

The patient underwent regular disease surveillance, including ophthalmological exams, total body and craniospinal MRI, and morphological and molecular (CRX mRNA) monitoring in BM and CSF during the first 2 years after the end of treatment (EoT). The patient remains alive and disease-free 10 years post initial diagnosis and 4 years after metastatic relapse. Current sequelae include ifosfamide-related nephropathy requiring magnesium supplementation, grade 1 hearing loss, and bilateral grade 4 vision loss.

### 2.2 Case 2

The second patient was diagnosed with bilateral RB at one year of age, with the left eye classified as group E and the right eye as group C according to the ICRB (35) (Figure 1B). The left eye was enucleated, and the right eye received conservative treatment. Initial therapy with systemic carboplatin, etoposide and vincristine (28, 29) included 15 cycles, achieving remission but leading to grade 3 hypoacusia.

Five years later, the patient presented with vitreous seeding in the right eye and was referred to our center. Comprehensive disease work-up, including craniospinal MRI, PET-FDG, BM examination, showed no extraocular dissemination and confirmed the presence of RB1 germline mutation. Intra-arterial chemotherapy to the right eye caused retinal detachment and neovascular glaucoma, necessitating enucleation. Histological studies revealed scleral infiltration, while BM examination demonstrated RB infiltration, and qRT-PCR detected CRX mRNA in the CSF. Systemic therapy followed the ARET0321, including induction chemotherapy and reduced intensity (thiotepa and etoposide only) myeloablative therapy and ASCT (18). Platins were omitted in both induction and myeloablative regimen due to prior ototoxicity. As CRX remained detectable in repeated CSF samples, 6 monthly cycles of intrathecal topotecan (0.4 mg) were administered, normalizing CRX levels after the first cycle. After achieving systemic and CNS CR, consolidation therapy with naxitamab and GM-CSF were administered with manageable toxicity, consisting of grade 2 pain and grade 1 urticarial reactions.

Patient underwent regular monitoring, including ophtalmological exams, craniospinal MRI, and molecular surveillance in BM and CSF. She remains alive and disease-free 8 years after diagnosis and 5 years post extraocular relapse. Current sequelae include total visual loss due to bilateral enucleation and moderate bilateral sensorineural hearing loss secondary to chemotherapy.

## 3 Discussion

We hereby report, two patients diagnosed with heritable RB receiving intensive, multimodal treatment over the course of their care. Following extraocular relapse and reduced intensity myeloablative therapy and ASCT, both patients were treated with GD2 mAbs and GM-CSF and remain disease-free over 4 and 5 years, respectively, after extraocular and CNS metastasic relapse.

Limited treatment options exist for extraocular RB. Although studies have shown that HDC-ASCT significantly improves survival (18), substantial acute and long-term complications, including toxic deaths and SMNs, highlight an urgent unmet medical need (18, 21).

Platinum-based systemic chemotherapy remains a cornerstone for patients with high-risk retinoblastoma (HR-RB) (18). However, platinum-related ototoxicity, associated with bilateral high-frequency sensorineural hypoacusia (14), compounds the disease-related visual impairment already affecting RB survivors, further reducing their quality of life (36). One of the reported patients underwent bilateral enucleation, resulting in complete vision, and developed severe hearing loss following systemic therapy. This precluded treatment with high-dose carboplatin to prevent further functional deterioration.

Targeted therapies aim to improve outcomes while reducing long-term side effects. Current and emerging research suggests a potential role for mAbs directed against GD2, a glycosphingolipid highly expressed during embryogenesis, and in developmental cancers such as NB and RB (Figure 1) (2, 37) Following extensive clinical development, several anti-GD2 mAbs have been integrated into first-line treatment for HR-NB (4–6, 38). Naxitamab, a humanized version of mu3F8, is FDA-approved for bone and bone marrow refractory/relapsed HR-NB (11).

Despite their proven efficacy in HR-NB, limited research exists on the role of anti-GD2 mAbs in RB. Preclinical experiments suggest that GD2-targeted therapy is effective against RB cell lines; however, there are few studies in the clinical setting. In a recent case series of 4 patients treated with the anti-GD2 mAb dinutuximab beta (ch14.18/CHO) after ASCT, Eichholz et al. demonstrated clinical responses in 2 patients with residual extraocular RB lesions after ASCT, radiotherapy and immunotherapy. However, the overlapping treatment timelines confounded the analysis of the individual contributions of each modality to these outcomes (39). Another report described a 9-year-old male with relapsed single bone metastatic RB treated with two cycles of dinutuximab beta with GM-CSF, aldesleukin (IL-2), and spironolactone after ASCT, demonstrating safety and no disease progression for up to 18 months post-treatment (40). Our experience provides further clinical evidence that long-term survival is achievable even with reduced chemotherapy intensity. While our findings do not support attributing the outcomes solely to naxitamab, it is notable that historical survival rates for metastatic RB patients before the advent of myeloablative chemotherapy were dismal (41, 42). Furthermore, in patients with recurrent metastatic extraocular RB treated with HDC and ASCT, 5-year survival rates remain at only 31.3% (43). Our results suggest that naxitamab may have contributed to the favorable outcomes observed in our patients, underscoring the potential of anti-GD2 immunotherapy to reduce the chemotherapy burden in extraocular RB.

Importantly, anti-GD2 mAbs exhibit acute and reversible toxicities but no long-term side effects have been reported to date. However, this observation is limited by the relatively short follow-up period since their approval. Additionally, as chemotherapy is typically administered prior to or alongside anti-GD2 therapy, overlapping toxicities may complicate the assessment of anti-GD2-specific effects in the future (44). In this case report, patients responded well to naxitamab, with adverse events consistent with previous reports and manageable using appropriate protocols (45, 46). Although preliminary, these results should guide future studies evaluating anti-GD2 mAbs and their potential to reduce reliance on highly toxic HDC-ASCT in RB patients.

Since anti-GD2 mAbs do not cross the BBB, patients with CNS metastasis also require CNS-targeted treaments, as CNS remains the primary site of RB metastatic relapse (16, 47). Recent reports support the use of intrathecal therapy to address CNS dissemination in RB, but timely detection remains challenging (20). Traditional methods, including CSF cytology and neuroimaging, only detect overt CNS disease, which is incurable (27, 48). Early detection and pre-emptive treatment of MDD, by contrast, may improve outcomes (30). Emerging evidence identifies CRX, a transcription factor upregulated in RB, as a specific biomarker for MDD (31, 32, 49). Although its prognostic role has yet to be confirmed (48), detectable CRX MDD in BM and CSF in non-metastatic RB patients with high-risk features is associated with decreased event-free survival (50) and CNS relapse (51), respectively. We routinely monitor CRX mRNA levels in the BM and CSF of high-risk RB patients. In this report, both patients achieved CR while successfully clearing CRX-positive MDD in the CSF using intrathecal topotecan. Continued monitoring has demonstrated no subsequent CNS progression. This approach underscores the importance of integrating molecular diagnostics with targeted CNS therapies to improve outcomes for high-risk RB patients.

Future therapeutic strategies may leverage CRX monitoring as a tool for early detection and to stratify patients who might benefit from intensified local CNS treatment, including novel combinations of intrathecal agents.

## 4 Conclusions

Our experience provides preliminary evidence for the potential role of anti-GD2 mAbs in the multimodality management of extraocular RB, especially for heavily pre-treated patients with significant cumulative prior toxicities and susceptibility to SMNs. Moreover, the results suggest that intrathecal topotecan may improve outcomes in patients with detectable CRX in the CSF. Taken together, the data support further investigation of anti-GD2 mAbs and CNS-directed therapy for MDD in well-defined subsets of patients with metastatic RB.

## Data Availability

The original contributions presented in the study are included in the article/Supplementary Material, further inquiries can be directed to the corresponding author.
